# Boid Inclusion Body Disease Is Also a Disease of Wild Boa Constrictors

**DOI:** 10.1128/spectrum.01705-22

**Published:** 2022-09-12

**Authors:** Alejandro Alfaro-Alarcón, Udo Hetzel, Teemu Smura, Francesca Baggio, Juan Alberto Morales, Anja Kipar, Jussi Hepojoki

**Affiliations:** a Departamento de Patología, Escuela de Medicina Veterinaria, Universidad Nacional, Heredia, Costa Rica; b Institute of Veterinary Pathology, Vetsuisse Faculty, University of Zurich, Zurich, Switzerland; c University of Helsinki, Faculty of Veterinary Medicine, Department of Veterinary Biosciences, Helsinki, Finland; d University of Helsinki, Faculty of Medicine, Medicum, Department of Virology, Helsinki, Finland; University of Prince Edward Island

**Keywords:** arenavirus, boa constrictor, boid inclusion body disease, inclusion body disease, veterinary microbiology, viral pathogenesis, wild snake

## Abstract

Reptarenaviruses cause boid inclusion body disease (BIBD), a potentially fatal disease, occurring in captive constrictor snakes boas and pythons worldwide. Classical BIBD, characterized by the formation of pathognomonic cytoplasmic inclusion bodies (IBs), occurs mainly in boas, whereas in pythons, for example, reptarenavirus infection most often manifests as central nervous system signs with limited IB formation. The natural hosts of reptarenaviruses are unknown, although free-ranging/wild constrictor snakes are among the suspects. Here, we report BIBD with reptarenavirus infection in indigenous captive and wild boid snakes in Costa Rica using histology, immunohistology, transmission electron microscopy, and next-generation sequencing (NGS). The snakes studied represented diagnostic postmortem cases of captive and wild-caught snakes since 1989. The results from NGS on archival paraffin blocks confirm that reptarenaviruses were already present in wild boa constrictors in Costa Rica in the 1980s. Continuous sequences that were *de novo* assembled from the low-quality RNA obtained from paraffin-embedded tissue allowed the identification of a distinct pair of reptarenavirus S and L segments in all studied animals; in most cases, reference assembly could recover almost complete segments. Sampling of three prospective cases in 2018 allowed an examination of fresh blood or tissues and resulted in the identification of additional reptarenavirus segments and hartmanivirus coinfection. Our results show that BIBD is not only a disease of captive snakes but also occurs in indigenous wild constrictor snakes in Costa Rica, suggesting boa constrictors to play a role in natural reptarenavirus circulation.

**IMPORTANCE** The literature describes cases of boid inclusion body disease (BIBD) in captive snakes since the 1970s, and in the 2010s, others and ourselves identified reptarenaviruses as the causative agent. BIBD affects captive snakes globally, but the origin and the natural host of reptarenaviruses remain unknown. In this report, we show BIBD and reptarenavirus infections in two native Costa Rican constrictor snake species, and by studying archival samples, we show that both the viruses and the disease have been present in free-ranging/wild snakes in Costa Rica at least since the 1980s. The diagnosis of BIBD in wild boa constrictors suggests that this species plays a role in the circulation of reptarenaviruses. Additional sample collection and analysis would help to clarify this role further and the possibility of, e.g., vector transmission from an arthropod host.

## INTRODUCTION

The first reports on boid inclusion body disease (BIBD) in captive snakes, mainly affecting members of the families *Boidae* and *Pythonidae*, go back to the 1970s (1). Others and ourselves identified novel arenaviruses as the causative agent(s) in the early 2010s (2–8), and in 2015, the BIBD-associated arenaviruses formed the genus *Reptarenavirus* in the family *Arenaviridae*, consequently leading to the formation of the genus *Mammarenavirus* (the previously known arenaviruses) (9). Until now, studies have described BIBD only in captive snakes, and the origin of reptarenaviruses remains unknown. However, our group recently confirmed the disease also in native captive boa constrictors in Brazil, providing evidence that reptarenaviruses may circulate in indigenous snake populations (10). As the name implies, BIBD manifests by the formation of eosinophilic and electron-dense cytoplasmic inclusion bodies (IBs) within almost all cell types (1, 11, 12). In fact, the antemortem diagnosis of BIBD relies on the detection of IBs in cells, for example, blood cells in cytological specimens of blood smears or hepatocytes in liver biopsy specimens (1, 13). More recently, the identification of reptarenavirus nucleoprotein (NP) as the main component of the IBs has allowed the use of immunohistochemistry to support the diagnosis, significantly increasing the diagnostic specificity and sensitivity (14, 15). Due to reasons unknown, IBs appear to be more common in reptarenavirus-infected boas than in pythons (2, 4, 7, 8, 13, 15). In naturally infected pythons, reptarenavirus infection predominantly manifested as central nervous system (CNS) signs which included regurgitation, “star-gazing,” head tremors, disorientation, and “corkscrewing”; these signs were also described in early reports of boas with BIBD but seem not to occur more recently (1). Indeed, experimental infection of pythons with reptarenavirus isolates induced CNS signs, whereas the infected boas remained without overt clinical signs (7, 8). In fact, different from the early reports on BIBD, the majority of boa constrictors with BIBD nowadays appear clinically healthy (15–17); however, other factors, such as circulating reptarenavirus species or concurrent infections, might contribute to the outcome of infection and the development of clinical disease.

Boas and pythons are nonvenomous constrictor snakes inhabiting biotopes of the neotropics and tropics. Boas occur in Central and South America and Madagascar, i.e., in the New World, whereas pythons occupy habitats in Africa, Asia, and Australia, i.e., in the Old World. Thus, the natural habitats of boas and pythons do not overlap much (The Reptile Database, http://reptile-database.org/). The literature has reported that several of the more than 100 known boa and python species are susceptible to BIBD (1).

Costa Rica harbors four indigenous boid species, namely, boa constrictor (Boa constrictor [18]), ringed or annulated tree boa (Corallus annulatus [19]), Central American or common tree boa (Corallus ruschenbergerii [19]), and rainbow boa (Epicrates cenchria [18]), distributed in different, partly overlapping habitats. Of these species, the common boa, annulated tree boa, and rainbow boa have been shown to be susceptible to BIBD (1, 15, 20). The boa constrictor (also known as red-tailed or common boa) has a wide distribution ranging from Florida (introduced species) to South America. In Costa Rica, it is distributed all over the country, inhabiting tropical and subtropical rain forests, versants, and Valle Central, at 0 to 1,500 meters above sea level. This nocturnal species lives mostly close to the ground and is often found close to coffee plantations, predominantly preying on mammals and birds (21). The annulated tree boa and the common tree boa are arboricole snakes. The annulated tree boa is distributed widely in Central America and Colombia. In Costa Rica, it inhabits the canopy of the Carribean subtropical and tropical rain forest, at 0 to 650 meters above sea level (21, 22). The common tree boa occupies habitats of the tropical rain forest of the Pacific south, i.e., Costa Rica, Panama, northern Colombia, northern Venezuela, Trinidad and Tobago, and Isla Margarita, at 0 to 500 meters above sea level (21, 22). The nocturnal rainbow boa has a wide distribution in Central and South America, from Costa Rica to Argentina, feeding predominantly on bats and rodents (23). In Costa Rica, it inhabits tropical rain forests, the Carribean versant, Pacific northwest, and Pacific south, at altitudes between 0 and 500 meters above sea level (21).

The family *Arenaviridae* currently comprises four genera, namely, *Mammarenavirus* (rodent- and bat-borne viruses), *Reptarenavirus* (the BIBD-associated viruses), *Hartmanivirus* (found in boa constrictors), and *Antennavirus* (fish viruses) (24, 25). The genome of reptarenaviruses has bisegmented negative-sense RNA with an ambisense coding strategy (24). The S segment encodes glycoprotein precursor (GPC) and nucleoprotein (NP), and the L segment gives rise to zinc finger matrix protein (ZP) and RNA-directed RNA polymerase (RdRp) (24). We identified Haartman Institute Snake virus 1 (HISV-1) during a sequencing study of snakes with BIBD (6), which after further characterization (26) became the founding member of the genus *Hartmanivirus* (27). HISV-1 and all other known hartmaniviruses lack the ZP gene (26) and do not appear to contribute to BIBD pathogenesis, although they often coexist with reptarenaviruses in snakes with BIBD (16, 26).

The literature best describes the occurrence of BIBD in captive snake collections in Europe, North America, Asia, and Australia (14–17, 28, 29). We recently reported BIBD and reptarenavirus infection in captive boa constrictors native to Brazil; however, the lack of detailed information on the origins of the studied snakes and their potential contacts during captivity did not allow us to conclude that reptarenaviruses are present in free-ranging (referred to as “wild” in the manuscript) snakes (10). Here, by studying captive and wild-caught native wild snakes with BIBD from Costa Rica, we show that BIBD occurs in wild boa constrictors, in conjunction with reptarenaviruses and hartmaniviruses.

## RESULTS

### Case descriptions.

In 2004 to 2006, we examined six captive snakes, namely, two annulated tree boas and four boa constrictors (Table 1, animals C1 to C6). The first snake (animal C1), a male annulated tree boa, had been wild caught and kept in captivity for 4 years, housed with another snake of the same species, before falling ill in 2004. It was euthanized due to necrotic lesions in the oral cavity. The postmortem examination revealed a fibrinonecrotic stomatitis with intralesional fungi and bacteria (identified in a direct smear) that remained unidentified, as a microbiological examination was not performed. The parasitological examination of gut content found *Eimeria* sp. oocysts, and the examination of a blood smear detected hemogregarine-infected red blood cells. The histological examination confirmed the gross findings and revealed the typical inclusion bodies (IBs) in parenchymal cells in various organs, including neurons in the brain, tubular epithelial cells in the kidney, hepatocytes, airway and lung epithelial cells, and pancreatic epithelial cells, as well as gastric, small and large intestinal epithelia (Table 1, animal C1). The blood smear that had been prepared immediately after the animal’s death showed the presence of IBs within erythrocytes.

**TABLE 1:** Information about animals included into the study^*a*^

Case no. and yr of examination	Animal data	Geographical site [further information]	Clinical sign	Pathological diagnosis	IB (confirmed by IH^*b*^)	NGS material and arenavirus segments identified
Captive animals						
C1, 2004	*C. annulatus,* male, 8.5 yr	Turrialba, Cartago [in captivity for 4 yr]	Necrotic stomatitis	Fibrinonecrotic stomatitis (mycotic), intestinal coccidiosis; hemogregarine infection (blood); BIBD	Brain +++; lung +; liver, kidney, pancreas, stomach ++; intestine +; testicle +; adrenal gland ++	FFPE (liver kidney, lung, intestine): Reptarenavirus segments, UGV/S6 S segment, ABV/L3 L segment
C2, 2006	*B. constrictor*, NR, juvenile (2 mo) [sibling of C3]	Heredia [privately owned small breeding colony; mortality in group of juveniles; two emaciated juveniles and their mother were examined]	Emaciation	Emaciation; BIBD	Brain ++	FFPE (brain): Reptarenavirus segments, UGV/S6 S segment, ABV/L3 L segment
C3, 2006	*B. constrictor*, NR, juvenile (2 mo) [sibling of C2]		Emaciation	Emaciation; BIBD	Brain, liver +; spleen ++	FFPE (brain, liver, spleen): Reptarenavirus segments, UGV/S6 S segment, ABV/L3 L segment
C4, 2006	*B. constrictor*, female, adult [mother of C2 and C3]		None; no gross lesions	BIBD	Brain, kidney ++; liver +	FFPE (brain, liver, spleen): Reptarenavirus segments, UGV/S6 S segment, ABV/L3 L segment
C5, 2006	*C. annulatus,* male, adult	Grecia, Alejuela	Vomiting, CNS signs^*c*^	Pyogranulomatous hepatitis, nephritis and splenitis; BIBD	Brain, lung, pancreas, liver, kidney, spleen, testicle +++; blood smear ++	ND
C6, 2006	*B. constrictor*, male, adult	Grecia, Alejuela	Abnormal head tilt	BIBD	Brain, pancreas, kidney ++; liver, spleen, intestine +; stomach −	ND
C7, 2018	*B. constrictor*, NR, adult (>9 yr)	Refugio Herpetologico Santa Ana [>9 yr in captivity]	None	BIBD [healthy animal; BIBD diagnosed as part of a health screen]	Pellet of tissue culture inoculated with blood ++	SNT (I/1Ki cells inoculated with blood sample): Reptarenavirus segments, UGV/S6 S segment, ABV/L3 L segment, PuVV-1/L4 segment, KePV/L15 L segment, GMV/L19 L segment, Hartmanivirus segments, UnNV-1 S segment, UnNV-1 L segment
Wild animals						
W1, 1989	*B. constrictor*, male, juvenile, 2 kg	Atlantic coast [wild caught]	Ulcerative stomatitis	Fibrinonecrotic stomatitis and tracheitis, fibrino-suppurative pneumonia; BIBD	IB in liver +; no IH reaction	FFPE (liver): Reptarenavirus segments, UGV/S6 S segment, ABV/L3 L segment
W2, 1989	*B. constrictor*, NR, juvenile	Central Valley [wild caught]	Poor body condition	Granulomatous dermatitis, hepatitis and coelomitis; BIBD	IB in liver +; no IH reaction	FFPE (liver): Reptarenavirus segments, UGV/S6 S segment, ABV/L3 L segment
W3, 2012	*B. constrictor*, female, adult	Heredia [wild caught]	Multiple traumatic injuries at head and body	Gout, endoparasitosis (*Kalicephalus* sp.), BIBD	Kidney +++; spleen ++; liver, pancreas +	FFPE (kidney, liver): Reptarenavirus segments, UGV/S6 S segment, ABV/L3 L segment
W4, 2018	*B. constrictor*, female, adult	Lagurilla de Heredia [wild caught]	Dehydration, ulcerative and necrotic dermatitis	Ulcerative stomatitis, systemic fungal infection; BIBD	Brain ++; pancreas +; intestine −	Frozen liver and SNT (I/1Ki cells inoculated with brain homogenate): Reptarenavirus segment, UGV/S6 S segment, ABV/L3 L segment, PuVV-1/L4 segment
W5, 2018	*B. constrictor*, NR, adult	Descamparaditos de Puriscal [wild caught in coffee plantation]	Stomatitis	Granulomatous stomatitis (fungal infection); BIBD	Brain, liver, kidney ++; pellet of tissue culture inoculated with brain ++	Frozen brain and SNT (I/1Ki cells inoculated with brain homogenate): Reptarenavirus segments, UGV/S6 S segment, ABV/L3 L segment, Hartmanivirus segments, BESV-1 S segment, BESV-1 L segment

a BIBD, boid inclusion body disease; IB, inclusion bodies; FFPE, formalin-fixed, paraffin-embedded tissue; IH, immunohistology; NR, not recorded; ND, not done; CNS, central nervous system; SNT, supernatant; UGV, University of Giessen virus; ABV, aurora borealis virus; PuVV, pura vida virus; KePV, keijut pohjoismaissa virus; GMV, gruetzi mitenant virus; UnNV-1, Universidad Nacional virus 1; BESV-1, big electron-dense squares virus 1.

b The presence and extent of IBs in the examined organs/tissues was scored semiquantitatively as follows: −, no IB detected; +, low prevalence and/or small Ibs; ++, medium prevalence; +++, high prevalence and/or large IBs.

c Supplemental material.

In 2006, diagnostic postmortem examinations identified five cases of BIBD in captive snakes. The owner of a small private breeding colony had observed high mortality in a group of juvenile captive-bred boa constrictors (10 of 27 died), due to which he submitted two emaciated snakes from the group (animals C2 and C3) for euthanasia and diagnostic postmortem examination. The examination did not reveal any other gross changes. The histological changes were restricted to the presence of abundant cytoplasmic IBs in the examined organs, i.e., neurons and ependymal cells in the brain, hepatocytes, and lymphocytes and erythrocytes in the spleen. After the BIBD diagnosis in the two juvenile animals and having been informed that reptarenavirus infection in the juveniles could be a consequence of vertical transmission from the parental animals, later shown in captive snakes by our group to occur (30), the owner requested euthanasia and a diagnostic postmortem examination of their mother (animal C4). Also in this animal, the pathological changes were restricted to the presence of cytoplasmic IBs in parenchymal cells of multiple tissues, namely, brain, liver, and kidney, thus confirming that the animal had also suffered from BIBD.

**FIG 1:**
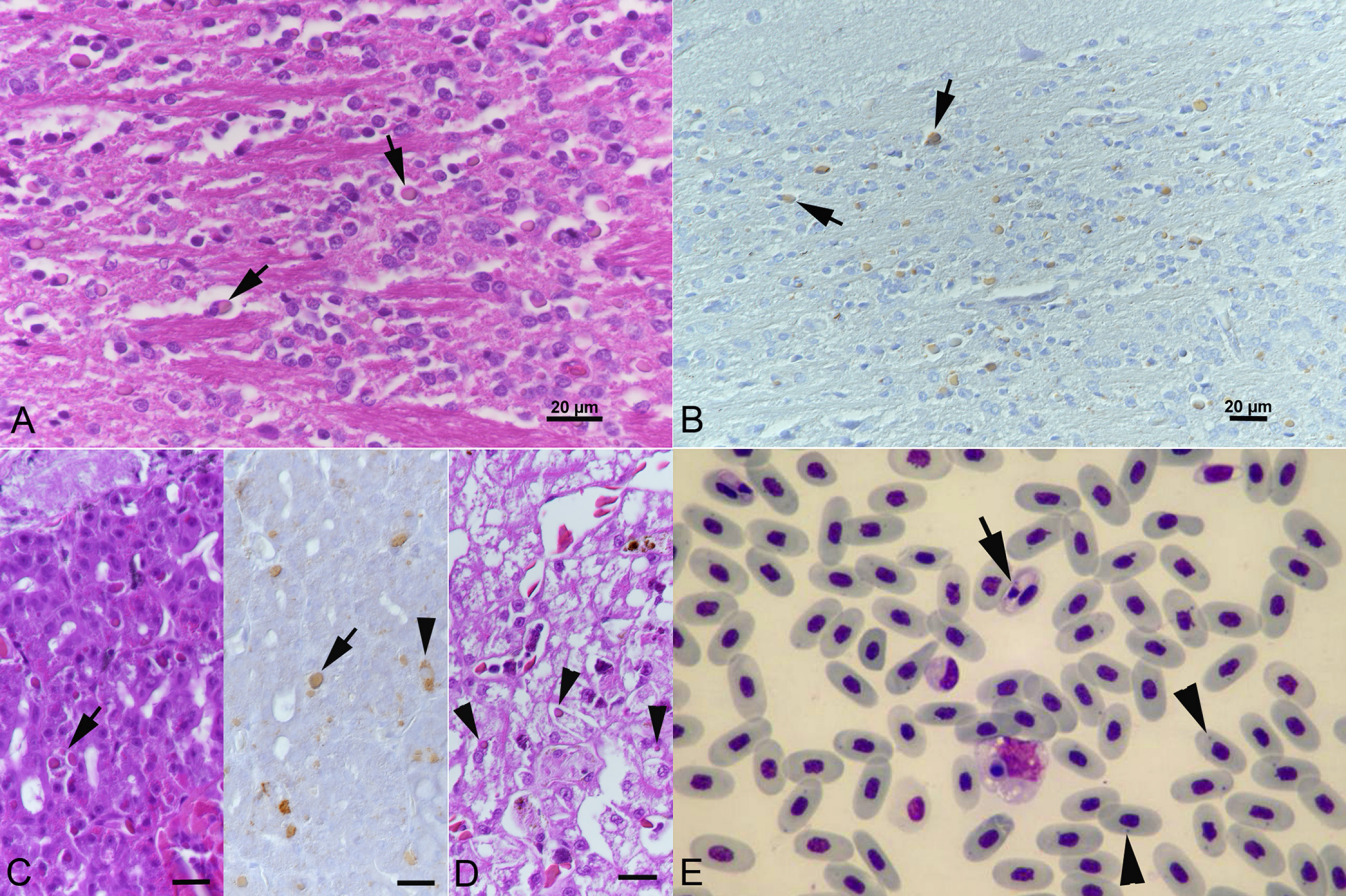
Animal C5, captive annulated tree boa (*C. annulatus*) with BIBD. (A, B) Brain stem. Cytoplasmic inclusion bodies (IBs) (arrows) in neurons that are comprised of reptarenavirus nucleoprotein (B). (C) Pancreas with IBs in epithelial cells (arrows). There are also individual cells with diffuse cytoplasmic viral antigen expression (right image, arrowhead). (D) Liver with IBs in hepatocytes (arrowheads). (E) Blood smear. Red blood cells with IBs (arrowheads) and hemogregarine infection (arrow). May-Grünwald-Giemsa stain. (A, C [left], and D) HE stain; (B, C [right]) immunohistology, hematoxylin counterstain. Bars = 20 μm.

Another two adult snakes, namely, a male annulated tree boa (animal C5) and a male boa constrictor (animal C6) from a private breeder, were euthanized due to central nervous system signs (for video, see Table 1). The diagnostic postmortem examination of the annulated tree boa revealed a pyogranulomatous hepatitis, nephritis, and splenitis and cytoplasmic IBs in parenchymal cells of various organs, i.e., the brain, lung, pancreas, liver, kidney, spleen, and testicle, and in blood cells (identified in a blood smear) (Fig. 1; Table 1, animal C5) which also showed hemogregarine infection of red blood cells (Fig. 1E). The boa constrictor did not exhibit any gross or histological changes apart from the presence of abundant cytoplasmic IBs in parenchymal cells of brain, pancreas, kidney, liver, spleen, and intestine (Table 1, animal C6).

The last animal of this series was a wild-caught boa constrictor (animal C7) that had been housed in captivity in a reptile park for over 9 years. The animal was one of several healthy snakes that underwent a health screen, including an examination of a blood smear to determine the BIBD status and detect or exclude protozoan infection, as a basis for new cohousing plans. The animal exhibited the typical cytoplasmic IBs in multiple blood cells (mainly erythrocytes) leading to the diagnosis of BIBD but showed no evidence of protozoan infection. It was subsequently housed in an individual terrarium remote from other snakes and was then lost for follow-up investigations.

The remaining five snakes of the study represent wild boa constrictors that had been caught by the local authorities. All were then euthanized by a veterinarian due to severe clinical disease. The first two animals were examined in 1989. A juvenile boa constrictor was caught at the Atlantic Coast (Table 1, animal W1) and found to suffer from a severe, focal extensive fibrinonecrotic and ulcerative stomatitis, a fibrinosuppurative tracheitis, and a moderate multifocal fibrinosuppurative bronchopneumonia with multiple microabscesses. The bacteriological examination isolated Escherichia coli (not further specified) from the lesions, and the parasitological examination revealed a moderate intestinal endoparasitosis (nematodes, not further specified). The second animal (Table 1, animal W2) had been found with poor body condition in the Central Valley. It was transferred to the national zoological garden where it did not feed during the quarantine period, and hence, it was euthanized. The postmortem examination revealed a multifocal ulcerative and pyogranulomatous to necrotic dermatitis with intralesional fungal structures (fungal culture, Fusarium spp.), as well as a multifocal granulomatous coelomitis (cranially to kidneys) and hepatitis. The animal also suffered from a moderate intestinal endoparasitosis (nematodes, not further specified). In both animals, the histological examination of the liver revealed the typical cytoplasmic IBs within hepatocytes, leading to the diagnosis of BIBD.

**FIG 2:**
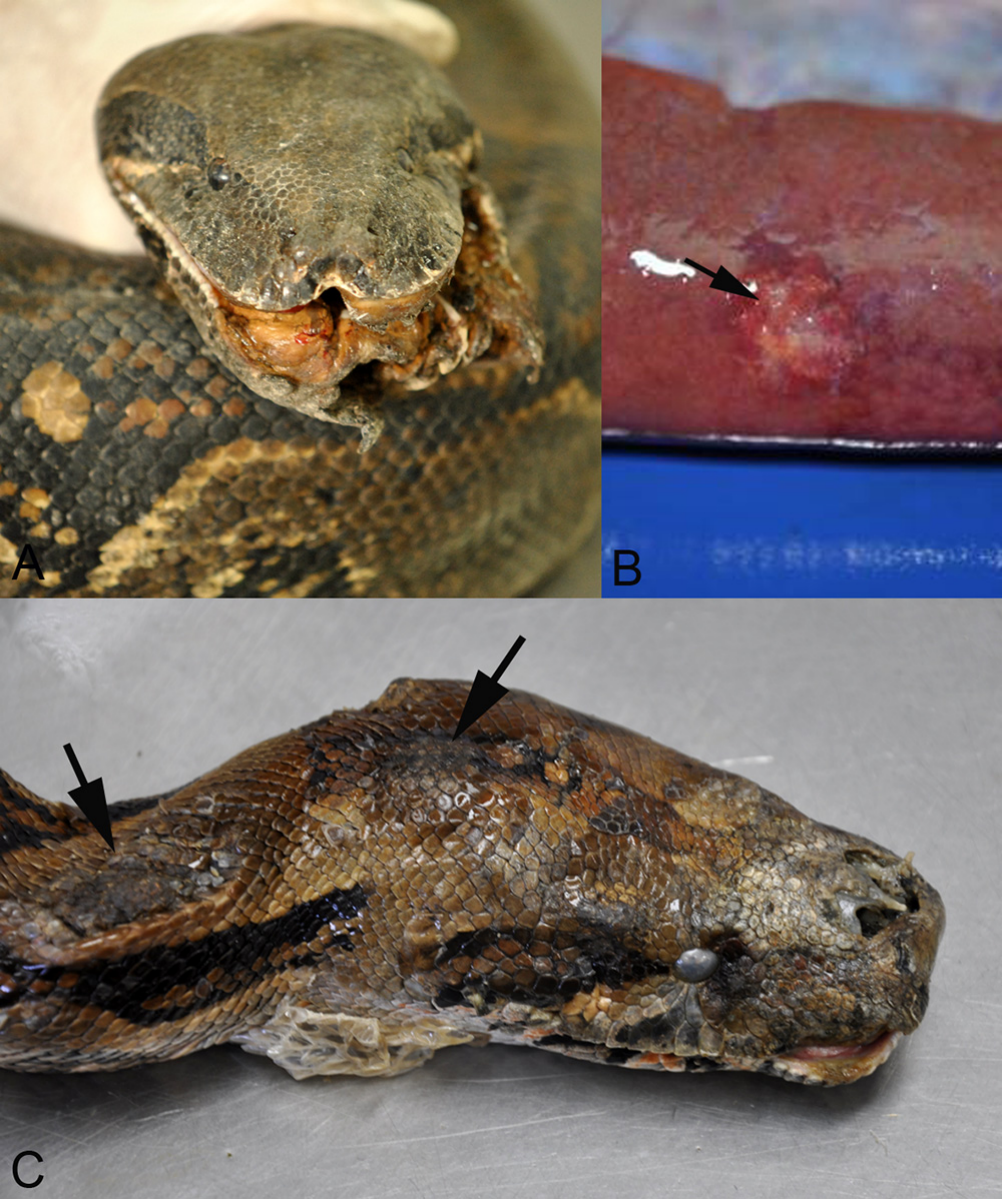
Gross findings in two wild-caught boa constrictors that were euthanized due to incurable disease. (A, B) Animal W3. (A) Head with severe traumatic injuries. (B) Liver with focal granulomatous inflammation in association with urate crystals (gout). (C) Animal W4. Head with multifocal necrotic dermatitis (arrows).

The third animal (Table 1, animal W3) was caught in 2012. It was euthanized due to multiple traumatic injuries of the head and body (Fig. 2A). The postmortem examination showed that the animal suffered from gout (Fig. 2B). The histological examination also revealed abundant cytoplasmic IBs in parenchymal cells of the kidneys, spleen, and liver.

**FIG 3:**
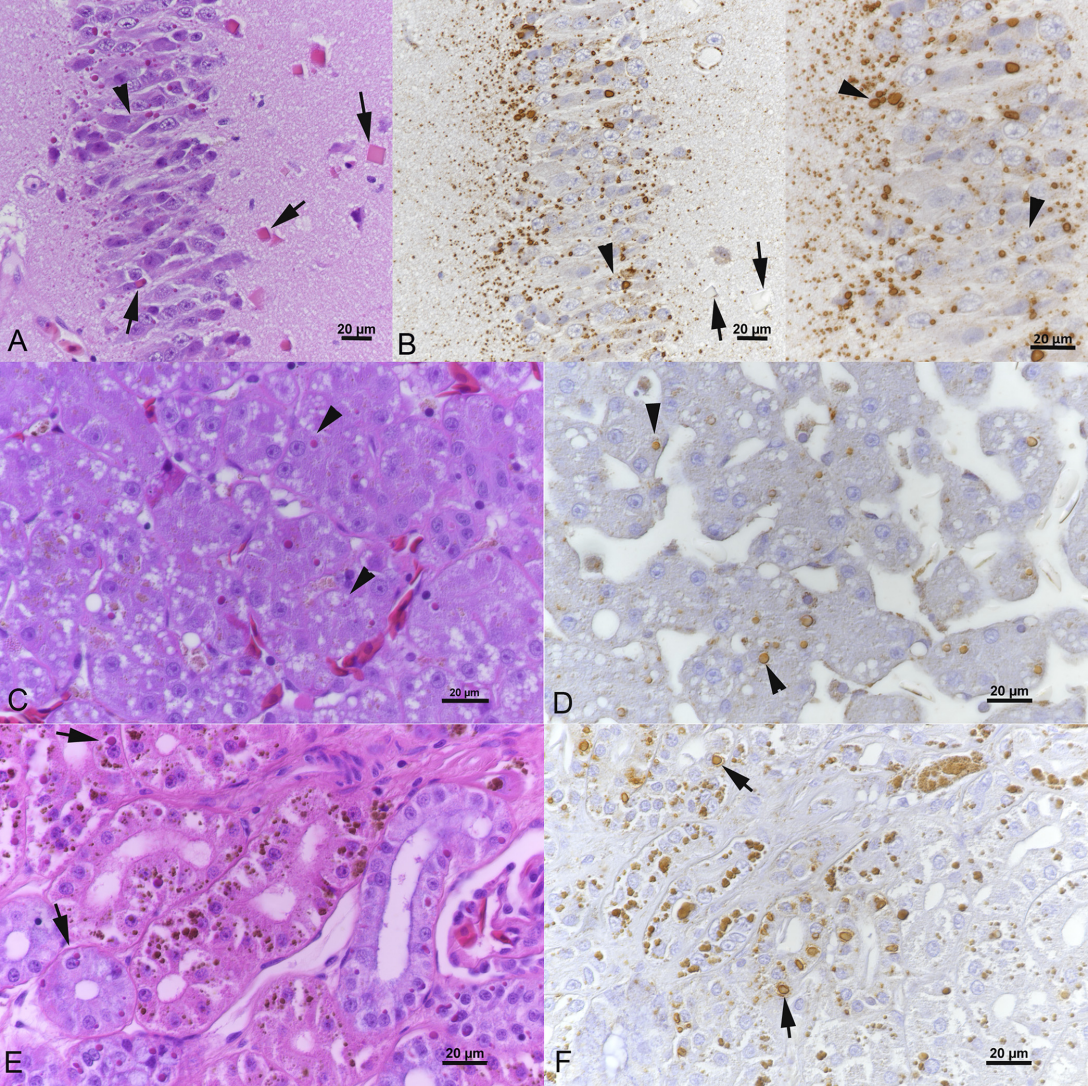
Wild-caught boa constrictor (animal W5) with BIBD. Inclusion body (IB) formation and reptarenavirus nucleoprotein (NP) expression in tissues. (A, B) Cerebral cortex with abundant cytoplasmic IBs in neurons. Some neurons exhibit more than one IB (arrowheads), and others show large square IBs (A, arrows) in which reptarenavirus NP expression is restricted to a focal punctate reaction (B, left image, arrows). (C, D) Liver with variably sized IBs in hepatocytes (arrowheads). (E, F) Kidney with variably sized IBs in tubular epithelial cells (arrows). Nota bene (NB): The coarse brown pigment in the epithelial cells represents lipofuscin-like degradation products frequently observed in the kidney of boas. (A, C, E) hematoxylin and eosin (H&E) stain; (B, D, F) immunohistology, hematoxylin counterstain. Bars = 20 μm.

In 2018, at a time when the group of investigators had started to collaborate to work up cases of BIBD in Costa Rica, another two wild-caught sick boas (Table 1, animals W4 and W5) were submitted for a diagnostic postmortem examination. The first animal was euthanized due to a multifocal necrotic dermatitis (Fig. 2C) and was found in the postmortem examination to have suffered also from an ulcerative stomatitis as well as a multifocal to coalescing heterophilic and granulomatous hepatitis, nephritis, and myocarditis with intralesional fungal structures (not further identified). The second animal was euthanized due to a granulomatous stomatitis with intralesional fungal structures (not further identified) without any other significant lesions. Both animals exhibited cytoplasmic IB in parenchymal cells in brain and pancreas and brain, liver, and kidney, respectively (Fig. 3). In the brain of animal W5, they were also obvious as large, brightly eosinophilic, partly square inclusions (Fig. 3A).

### Confirmation of BIBD and reptarenavirus infection by immunohistology.

In 2021, immunohistology for the detection of reptarenavirus NP was performed on tissue sections from all snakes. This assay yielded a positive result (Fig. 1B and C), thereby confirming reptarenavirus infection in all cases apart from the two wild snakes from 1989, of which the paraffin blocks had likely suffered from protein degradation during storage (Table 1).

### Virus isolation.

**FIG 4:**
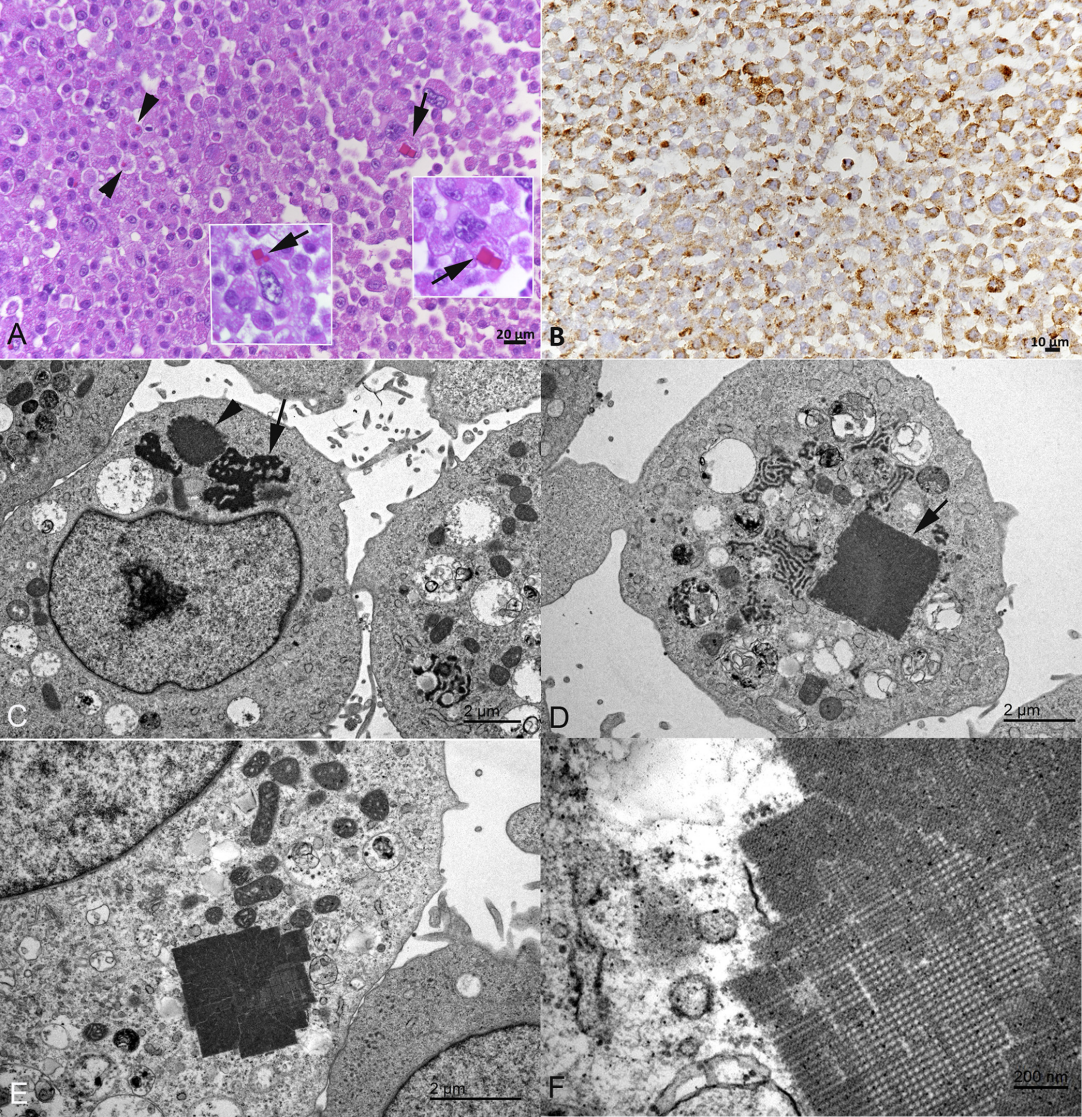
Wild-caught boa constrictor (animal W5) with BIBD. Light and transmission electron microscopy findings in a cell pellet prepared from the boa constrictor (*B. constrictor*) kidney-derived cell line I/1Ki at 6 days postinoculation with brain homogenate. (A, B) Inclusion body (IB) formation (A, arrowheads) and reptarenavirus nucleoprotein (NP) expression (B) in the cells. Like the neurons in the cerebral cortex (Fig. 3A), individual cells exhibit large square IBs (arrows). (C to F) Ultrastructural features. The cells exhibit smaller and irregular early (arrow) and more complex older (arrowhead) IBs (C) and occasional large square IBs (D) with a grid-like, directed structure (E, F).

Virus isolation was attempted from three boa constrictors examined in 2018 (Table 1). The blood from captive snake C7, a liver homogenate of wild-caught snake W4, and a brain homogenate of wild-caught snake W5 served to inoculate I/1Ki (*B. constrictor* kidney cell line) cell cultures. Immunohistology of cell pellets prepared after 6 days of inoculation showed extensive inclusion body formation and reptarenavirus NP expression in all inoculated cells (Fig. 4B). Hartmanivirus NP expression was not observed. Interestingly, like neurons in the brain of the affected snake, the cultures inoculated with the brain homogenate of animal W5 exhibited occasional large, brightly eosinophilic, square cytoplasmic inclusions (Fig. 4A) interpreted as overwhelming NP expression. Ultrastructurally, the cells were found to harbor the previously described early and late inclusions as well as dense, large square inclusions with a grid-like electron dense pattern (Fig. 4C to F) reminiscent of protein crystals.

### Identification of viruses.

**FIG 5:**
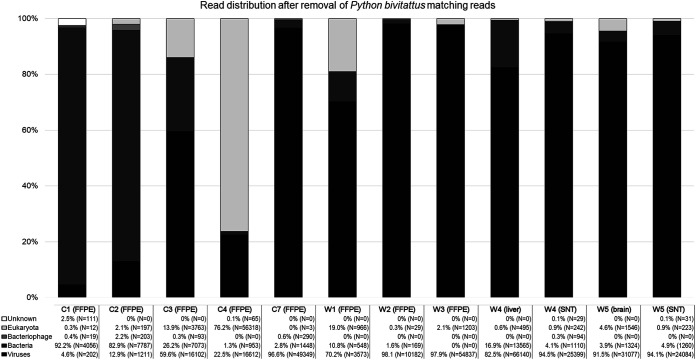
The distribution of reads in Lazypipe (33) analysis after the removal of reads matching to *Python bivitattus* genome. The *y* axis represents percentage of reads. The reads matching viruses, bacteria, bacteriophages, eukaryota, and unknown (i.e., those not without match) are shown in colors ranging from black to white as indicated in bottom left corner. The number of reads matching each category are presented in written format (% of total reads of the sample and the number of reads in brackets) under each respective sample name.

To confirm reptarenavirus infection and to identify the infecting viruses, we performed a metatranscriptomic analysis of the formalin-fixed paraffin-embedded (FFPE) archival material (Table 1, animals C1 to C4 and W1 to W3), freshly frozen liver (Table 1, animal W4) and brain (Table 1, animal W5), and supernatants of cell cultures inoculated with tissue homogenates and blood (Table 1, animals C7, W4, and W5), respectively. In addition to the *de novo* genome assembly-based approach utilized in our earlier studies (6, 10, 16, 26, 30–32), we used the lazypipe NGS pipeline for pathogen discovery (33). The lazypipe served to produce an overview of the microbial reads in the samples after removal of the host genome from the data set, demonstrating the presence of mainly viruses and bacteria (Fig. 5). Most of the eukaryotic reads remaining in the samples represented members of the families Aspergillaceae and *Thermoascaceae*, possibly representing the causative agent of the mycotic disease and/or reflecting contamination and deterioration of the archival paraffin blocks. Additionally, lazypipe identified some reads matching potential prey animals (families *Passerellidae*, *Sylviidae*, *Phasianidae*, and *Columbidae*) and blood-sucking parasites (family *Argasidae* and *Trombidiidae*).

The viruses identified through the lazypipe analysis of the samples that went through NGS are summarized in Table 2. The majority of the viral reads not matching arenaviruses included viruses (families *Partitiviridae*, *Chrysoviridae*, and *Botourmiaviridae*) infecting fungi, plants, and possibly insects but also viruses (families *Adenoviridae*, *Picobirnaviridae*, and *Nodaviridae*) that may indeed have infected the studied animals. The analysis confirmed reptarenavirus infection in all of the studied animals and identified hartmanivirus infection in two animals (Table 1, animals C7 and W5). For most samples, the standard lazypipe run did not produce complete segment-length contigs, and we employed MIRA assembler (http://mira-assembler.sourceforge.net/docs/DefinitiveGuideToMIRA.html) as well as Bowtie 2 (34) to assemble full-length arenavirus S and L segments. As presented in Table 1, all animals studied carried the University of Giessen virus (UGV) S segment and aurora borealis virus 3 (ABV-3) L segment, i.e., S6 and L3 segments according to the nomenclature coined by Stenglein et al. (5). Two of the studied animals (Table 1, C7 and W4) showed the presence of more than a single pair of reptarenavirus S and L segments, and both harbored an L4 L segment earlier identified by Stenglein and colleagues (5), which we designated the L segment of pura vida virus 1 (PuVV-1). The analysis revealed two additional L segments, i.e., those of keijut pohjoismaissa virus 1 (KePV-1) and gruetzi mitenant virus 1 (GMV-1), respectively, known as L15 and L19 following the Stenglein et al. (5) nomenclature, in the animal C7. All of the identified reptarenavirus segments were highly similar to those described in earlier studies.

**TABLE 2:** Distribution of NGS reads matching to viruses as identified by Lazypipe (33)

Animal no. (material)	No. of reads	No. of contigs	Virus family	Genus
C1 (FFPE: liver, kidney, lung, intestine)	195	3	*Arenaviridae*	*Reptarenavirus*
	7	0	*Picobirnaviridae*	Unknown
C2 (FFPE: brain)	1,090	0	*Partitiviridae*	Unknown
	85	3	*Adenoviridae*	*Mastadenovirus*
	32	1	*Nodaviridae*	*Alphanodavirus*
	4	1	*Arenaviridae*	*Reptarenavirus*
C3 (FFPE: brain, liver, spleen)	15,921	6	*Arenaviridae*	*Reptarenavirus*
	64	0	*Partitiviridae*	Unknown
	37	0	*Picobirnaviridae*	Unknown
	33	0	*Hypoviridae*	Unknown
	17	0	Unknown	Unknown
	13	2	*Adenoviridae*	*Mastadenovirus*
	13	2	*Botourmiaviridae*	*Ourmiavirus*
	4	1	*Chrysoviridae*	*Betachrysovirus*
C4 (FFPE: brain, liver, spleen)	9,063	0	Unknown	Unknown
	2,463	5	*Chrysoviridae*	*Chrysovirus*
	1,868	5	*Botourmiaviridae*	*Ourmiavirus*
	1,340	0	*Partitiviridae*	Unknown
	791	1	*Partitiviridae*	*Gammapartitivirus*
	528	3	*Chrysoviridae*	*Betachrysovirus*
	340	0	*Picobirnaviridae*	Unknown
	199	3	*Arenaviridae*	*Reptarenavirus*
	18	1	*Chrysoviridae*	*Alphachrysovirus*
	2	1	*Endornaviridae*	*Alphaendornavirus*
C7 (cell culture medium)	49,006	132	*Arenaviridae*	*Reptarenavirus*
	343	6	*Arenaviridae*	*Hartmanivirus*
W1 (FFPE: liver)	3,570	7	*Arenaviridae*	*Reptarenavirus*
	3	1	*Adenoviridae*	*Mastadenovirus*
W2 (FFPE: liver)	10,182	10	*Arenaviridae*	*Reptarenavirus*
W3 (FFPE: tissues)	54,831	8	*Arenaviridae*	*Reptarenavirus*
	6	1	*Retroviridae*	*Gammaretrovirus*
W4 (frozen liver)	66,140	200	*Arenaviridae*	*Reptarenavirus*
W4 (cell culture medium)	25,399	18	*Arenaviridae*	*Reptarenavirus*
W5 (frozen brain)	29,780	275	*Arenaviridae*	*Reptarenavirus*
	1,297	2	*Arenaviridae*	*Hartmanivirus*
W5 (cell culture medium)	23,786	11	*Arenaviridae*	*Reptarenavirus*
	298	7	*Arenaviridae*	*Hartmanivirus*

**FIG 6:**
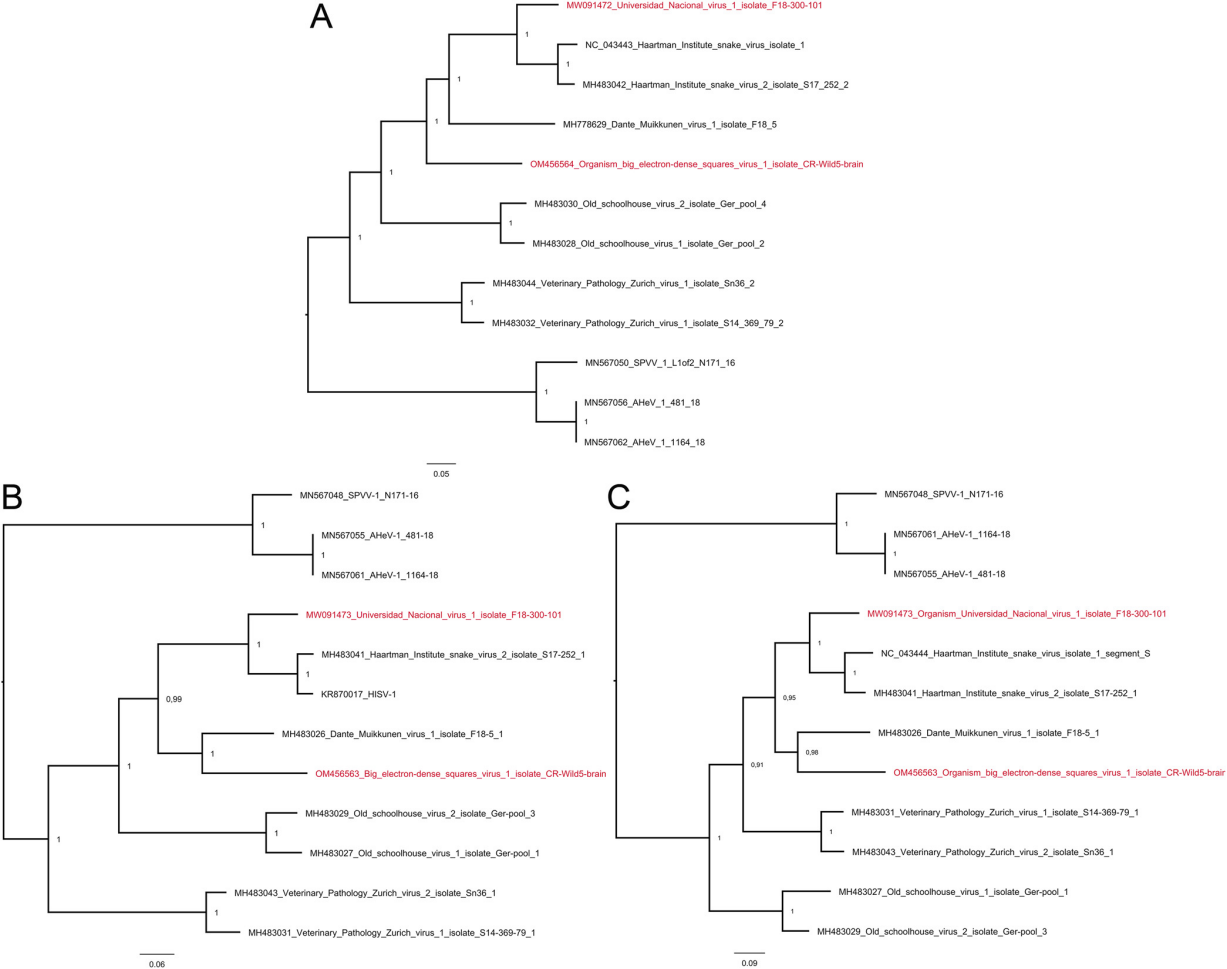
Phylogenetic analysis of hartmaniviruses identified in the study. The maximum clade credibility trees were inferred using the Bayesian MCMC method with Cprev Blosum and WAG amino acid substitution models for RdRp, GPC, and NP, respectively. (A) A phylogenetic tree based on the RdRp amino acid sequences of the viruses identified in this study and those available in GenBank. (B) Phylogenetic tree based on the NP amino acid sequences of the viruses identified in this study and those available in GenBank. (C) A phylogenetic tree based on the GPC amino acid sequences of the viruses identified in this study and those available in GenBank.

The NGS approach led to the identification of two pairs of hartmanivirus S and L segments, namely, one in animal C7 and the other in animal W5. Pairwise sequence comparison (PASC) analysis (35) of the hartmanivirus segments found in animal C7 animal showed that the S segment has 75.61% nucleotide identity to Haartman Institute snake virus 1 (HISV-1) S segment and that the L segment has 77.75% identity to the L segment of HISV-1. Similarly, the hartmanivirus S segment found in animal W5 has 67.06% identity to Dante Muikkunen virus 1 (DaMV-1) S segment, whereas the L segment had 68.51% identity to the L segment of DaMV-1. We further compared the identified hartmanivirus segments to those available from GenBank by generating a similarity matrix (Table 3 and 4). According to the PASC analysis and the distance matrix, the hartmanivirus L segment identified from the samples of animal C7 could be classified into the same species with HISV-1 and -2 if strictly following the current arenavirus taxonomic demarcation criteria (9). However, the corresponding analyses for the S segment would support the generation of a novel hartmanivirus species, due to which we named the corresponding virus as Universidad Nacional virus 1 (UnNV-1). Both PASC and similarity matrix analyses of the S and L segments identified in animal W5 supported the generation of a novel hartmanivirus species. We named the respective virus as big electron-dense squares virus (BESV-1) due to the presence of large square-shaped electron-dense accumulations observed in electron microscopy of BESV-1-infected cells. We further performed phylogenetic analysis of the hartmanivirus S and L segments (Fig. 6), which supports the classification of these viruses as novel hartmanivirus species. Specifically, on the basis of RdRp, UnNV-1 forms a sister clade for HISV-1 and HISV-2, whereas BESV-1 forms a sister clade for all DaMV-1, UnNV-1, HISV-1, and HISV-2 (Fig. 6A). Congruently with the similarity matrices, BESV-1 clustered together with DaMV-1 on the basis of GPC and NP, and they formed a sister clade for UnNV-1, HISV-1, and HISV-2 (Fig. 6B and C).

**TABLE 3:** Comparison of hartmanivirus L segments identified this study to full-length sequences available from GenBank^*a*^

Virus, GenBank accession no.	Nucleotide identity (%) of:										
	AHeV-1	AHeV-1	HISV-1	HISV-2	OScV-1	OScV-2	VPZV-1	VPZV-2	DaMV-1	UnNV-1	BESV-1
AHeV-1, MN567056.1											
AHeV-1, MN567062.1	94										
HISV-1, NC_043443.1	56	59									
HISV-2, MH483042.1	57	60	89								
OScV-1, MH483028.1	56	59	65	65							
OScV-2, MH483025.1	56	59	65	65	85						
VPZV-1, MH483040.1	57	60	65	65	65	65					
VPZV-2, MH483044.1	56	59	65	65	64	64	86				
DaMV-1, MH778629.1	56	59	69	69	65	65	65	65			
UnNV-1, MW091472.1	57	60	78	79	65	66	65	65	69		
BESV-1, OM456564	56	59	69	69	66	66	66	65	70	69	

a The numbers represent nucleotide identities (%) between L segments; light gray indicates >76% nucleotide identity (suggesting classification within same species [9]). AHeV, andere heimat virus; HISV, Haartman Institute snake virus; OScV, old schoolhouse virus; VPZV, veterinary pathology Zurich virus; DaMV, Dante Muikkunen virus; UnNV, Universidad Nacional virus; BESV, big electron-dense squares virus.

**TABLE 4:** Comparison of hartmanivirus S segments identified in this study to full-length sequences available from GenBank^*a*^

Virus, GenBank accession no.	Nucleotide identity (%) of:											
	SPVV-1	AHeV-1	AHeV-1	HISV-1	HISV-2	OScV-1	OScV-2	VPZV-1	VPZV-2	DaMV-1	UnNV-1	BESV-1
SPVV-1, MN567048.1												
AHeV-1, MN567055.1	76											
AHeV-1, MN567061.1	75	98										
HISV-1, NC_043444.1	53	52	53									
HISV-2, MH483041.1	54	53	54	86								
OScV-1, MH483027.1	53	52	53	60	60							
OScV-2, MH483029.1	54	52	52	61	61	78						
VPZV-1, MH483039.1	52	51	52	61	60	60	58					
VPZV-2, MH483043.1	52	51	52	62	61	59	58	84				
DaMV-1, MH483026.1	52	52	53	64	64	60	61	61	61			
UnNV-1, MW091473.1	53	53	54	75	74	61	60	61	61	66		
BESV-1, OM456563.1	53	53	53	65	65	61	60	61	61	68	66	

a Light gray indicates >80% nucleotide identity (suggesting classification within same species [9]). AHeV, andere heimat virus; HISV, Haartman Institute snake virus; OScV, old schoolhouse virus; VPZV, veterinary pathology Zurich virus; DaMV, Dante Muikkunen virus; UnNV, Universidad Nacional virus; BESV, big electron-dense squares virus.

## DISCUSSION

The initial descriptions of BIBD date back to the 1970s and have reported the disease only in captive snakes (1). Prior to the identification of reptarenaviruses as the causative agent (2–4, 7, 8), reports suggested that the disease can affect a multitude of snake species (1). The studies on reptarenaviruses have focused on captive constrictor snakes; it is so far unknown whether reptarenaviruses occur in wild animals and in different snake species as natural hosts. In a previous study, we provided evidence of reptarenavirus infections in native Brazilian boa constrictors by studying diseased snakes from wild animal sanctuaries; however, we could not rule out the possibility of reptarenavirus infection/transmission during captivity (10). Here, we report that BIBD occurs in indigenous captive snakes and wild boa constrictors in Costa Rica, and by studying archival tissue blocks, we demonstrate that the disease and its causative viruses have been present in wild boa constrictors since at least the late 1980s.

The clinical signs and pathological findings in the wild boa constrictors are comparable to those of “classical” BIBD in the early descriptions of the disease in captive boas (11, 12). Interestingly, in our experience, similar findings have been rare among captive boas in Europe since the beginning of the new millennium. In the present study, both wild and captive boa constrictors were found to have BIBD, and in annulated tree boas, the disease was restricted to captive individuals. This finding could suggest that reptarenavirus infection was transmitted to the annulated tree boas, directly or indirectly, from an infected boa constrictor or other animals of the collection. The samples collected from the animals (C7, W4, and W5) euthanized in 2018 enabled us to attempt virus isolation in cell culture. We performed metatranscriptomic analysis of both the tissue used for the inoculation of kidney-derived boa constrictor cells (I/1Ki cell line, described in reference 4) and the cell culture supernatant collected from the infected cells. For animals W4 and W5, we recovered an identical set of reptarenavirus and/or hartmanivirus L and S segments in both samples. While the metatranscriptomic sequencing of the blood sample of animal C7 failed, we could detect four reptarenavirus L segments, one reptarenavirus S segment, and a pair of hartmanivirus segments in the cell culture supernatant. The results of the inoculation experiments imply, as suggested also by our earlier studies, that I/1Ki cells are highly permissive for reptarena- and hartmaniviruses. In fact, one of the main motivational drivers for attempting virus isolation in the present study was to amplify the reptarenavirus segments for sequencing. Due to the presence of multiple segments and potential segment pairs in the isolates and the fact that our primary goal was to demonstrate the presence of reptarenaviruses in wild boas, we did not attempt producing multistep growth curves for the isolates. While not a focus of the present study, the growth kinetics and pairing of the isolated L and S segments merit further investigations.

The association of each mammarenavirus species with a specific rodent (or bat) host has led to the hypothesis of virus-host coevolution (36). The current knowledge on reptarenaviruses presents a very different scenario in which the majority of snakes with BIBD carry several L and S segments of different reptarenavirus species (5, 6, 10, 16, 30). We cannot exclude that a sampling bias (examination of captive snakes with BIBD, and mainly boa constrictors) can explain the observed frequent coinfections. Snakes with BIBD often carry more L segments (we have identified up to seven in one snake [30]) than S segments (we usually find 1 to 3 different S segments in one animal). One could speculate that the frequent reptarenavirus coinfections seen in captive snakes (5, 6, 10, 16, 30) are a result of cross-species transmission of reptarenaviruses between snakes of different species and/or origin during transportation or cohousing. Reptarenavirus infection in boa constrictors is vertically transmitted from both the sire (father) and the dam (mother) (30). This transmission could have allowed the accumulation of L and S segment pools over a relatively long time (the first descriptions of BIBD are from the 1970s). The wild boa constrictors of this study appeared to carry only a single pair of L and S segments. Interestingly, the S segment found in these animals represents the same species as University of Giessen virus (UGV; or S6 segment following the nomenclature of Stenglein et al. [5]) which is at present the S segment most often found in captive boa constrictors with BIBD (5, 16, 30). If snakes were the reservoir hosts of reptarenaviruses, one could hypothesize that this particular S segment belongs to the reptarenaviruses that coevolved with the boa constrictor. However, none of the animals included in our earlier study describing reptarenaviruses in native Brazilian boa constrictors carried the UGV-/S6-like S segment (10), thus negating the hypothesis. One could further hypothesize that the UGV-/S6-like segments seen in captive snakes in United States and Europe would originate from wild-caught snakes in the Middle American region. The results also raise the general question as to whether a virus of which boas are the natural host would lead to BIBD, a disease that is eventually fatal, perhaps due to associated immunosuppression (1). If the reptarenaviruses found in the wild animals of this study are not native to boa constrictors, then how did the animals acquire the infection? Cross-species transmission from the prey animals of the snakes represents one option for acquiring the infection. The diet of wild boa constrictors in Costa Rica consists of a wide range of animals, including birds, mammals (e.g., mice, rats, possums, and bats), lizards, and iguanas. It is basically identical in Brazil, and different prey species could carry different reptarenaviruses, potentially explaining the wide variety of reptarenaviruses identified in snakes. However, using cultured cells of different animal species, we demonstrated earlier that reptarenavirus replication occurs at 30°C rather than at 37°C (37), which suggests the potential natural host to be cold-blooded/heterothermic. It would thus also be possible that boa constrictors acquire the infection through bites of blood-sucking parasites. In line with this arthropod transmission hypothesis, anecdotal evidence suggests that snake mite infestation of snake colonies coincides with the occurrence of BIBD (1). Furthermore, we have demonstrated that reptarenaviruses can infect cultured cells of three different tick species (37). Given the potential broad host range of the viruses, there is a need for further studies over a wide range of wild animals, including snakes, to identify the reservoir host of reptarenaviruses.

Like reptarenaviruses, the origin of hartmaniviruses remains a mystery. We have identified several hartmaniviruses during our earlier NGS studies on snakes with BIBD (6, 16, 26, 32); however, unlike reptarenaviruses, hartmaniviruses appear to always be present as a single pair of S and L segments. So far, we have not associated hartmanivirus infection with any pathological findings or clinical signs, but neither have we been able to rule out their pathogenic potential. The present study identified two novel hartmaniviruses, namely, UnNV-1 and BESV-1, and we managed to cultivate both viruses in boid kidney cells, albeit in coinfection with reptarenaviruses. Interestingly, the cultures with BESV-1 displayed large inclusions that were brightly eosinophilic in the hematoxylin-eosin stain. Ultrastructural analysis of the infected cells revealed the presence of large square-shaped electron-dense inclusions within the infected cells. The reptarenavirus S segment present in a coinfection with BESV-1 hartmanivirus is classified into *Giessen reptarenavirus* species, which is the most commonly found reptarenavirus S segment in snakes with BIBD (5, 16). It is thus unlikely—and supported by our immunohistological findings—that the square-shaped inclusions observed in the infected cells would be associated with reptarenavirus NP expression. This idea could suggest that BESV-1 infection can induce IB formation, perhaps via NP expression, and would thus represent the first hartmanivirus associated with IB formation.

## MATERIALS AND METHODS

### Animals.

The studied animals included 11 snakes submitted to the Department of Veterinary Pathology, Universidad Nacional, Heredia, Costa Rica, for diagnostic postmortem examination. Among them were six captive snakes, four boa constrictors (*B. constrictor*), and two annulated tree boas (*C. annulatus*), examined in 2004 and 2006 (animals C1 to C6), and five wild boa constrictors that were caught and immediately euthanized due to severe clinical disease in 1989, 2012, and 2018 (animals W1 to W5) (Table 1). Due to the unclear taxonomic classification of *B. constrictor* subspecies (38, 39), we only refer to boa constrictors in the manuscript. From all animals, specimens from selected organs and tissues were collected at necropsy and were fixed in 10% buffered formalin for histological examination. From the snakes examined in 2018, additional tissue samples (brain and liver) were collected and frozen at −80°C for virus isolation and RNA extraction.

The study also included one apparently healthy boa constrictor from a reptile sanctuary that the owners wished to be screened for BIBD and hemoparasites before moving it to housing with closer contact to other snakes. From this animal (C7), a blood sample was collected in a 1.3-mL K3E EDTA tube (Sarstedt) by venipuncture of the caudal tail vein, aliquoted (one sample diluted 1:1 in RNAlater [ThermoFisher Scientific], one direct), and frozen at −80°C for RNA extraction and virus isolation, respectively. In addition, blood smears were prepared.

No ethical permissions were required for these diagnosis-motivated blood and tissue samplings, which were requested by the owners and permitted by the authorities, respectively. To specify, all postmortem examinations of wild snakes were conducted with approval by the Ministry of Environment and Energy (MINAE) (wildlife authority) through permit R-SINAC-PNI-ACLAC-039, and with the support of the animal health authority, the National Animal Health Service through the permit SENASA-DG-0277-18.

### Histology, immunohistology, and cytology.

Formalin-fixed tissue specimens were trimmed and embedded in paraffin wax using routine methods. Sections (3 to 5 μm) were prepared at the time of diagnosis and stained with hematoxylin-eosin (HE). At a later stage, in 2021, consecutive sections were subjected to immunohistological staining for reptarenavirus NP as described (4, 10). For cytological examination of blood cells for the presence of inclusion bodies, the current standard antemortem diagnostic approach (1, 15), blood smears taken from animals C1, C5, and C7 were stained with May-Grünwald-Giemsa and examined by light microscopy as described (16, 30).

### Cell culture and virus isolation.

The boa constrictor kidney-derived cell line I/1Ki, maintained at 30°C and 5% CO_2_, was used for virus isolation attempts as described (4). Tissue cubicles of 1 to 2 mm^3^ of liver (animal W4) and brain (animals W5) were defrosted, mechanically homogenized using a razor blade and up-down pipetting in 6 to 8 mL of fully supplemented growth medium (minimal essential medium Eagle [Sigma], 10% of fetal bovine serum [Gibco], 10% of tryptose-phosphate broth [Sigma-Aldrich], 2 mM l-glutamine, 1.4 mM HEPES buffer, and 100 IU/mL gentamicin), clarified by centrifugation (5 min and 300 relative centrifugal force [RCF]) at 10°C, and filtered through a 0.45-μm syringe filter (Millipore). Confluent layers of I/1Ki cells in 75-cm^2^ culture flasks were inoculated with 1 mL of filtered inoculum for 6 to 12 h, followed by a media exchange.

For virus isolation from the blood (animal C7), we inoculated a subconfluent culture of I/1Ki cells with 300 μL of blood diluted in 3 mL fully supplemented growth medium filtered through a 0.45-μm syringe filter. After 4 h adsorption at 30°C and 5% CO_2_, 4 mL of fresh medium was added and the culture incubated overnight at 30°C and 5% CO_2_. The following day, we washed the cells once with fresh medium and continued the incubation at 30°C and 5% CO_2_.

For all cultures, the cells were kept for 6 days and the media exchanged after 3 days. Supernatants from both time points were collected, mixed, and frozen at −80°C. For the preparation of cell pellets from the inoculated cell cultures, cells were trypsinised (trypsin 10×; Merck Biochrome) for 5 min at room temperature and then spun at 1,000× RCF for 3 min. The resulting pellet was either fixed in 4% buffered paraformaldehyde overnight and routinely embedded in paraffin wax for histology and immunohistology or was fixed in 2.5% phosphate-buffered glutaraldehyde and embedded in epoxy resin for transmission electron microscopy using routine methods, as described previously (26, 40).

### Next-generation sequencing (NGS) and genome assembly.

RNAs were extracted from paraffin-embedded tissue samples of animals C1 to C4 and W1 to W3 (Table 1) using the RNeasy FFPE kit (Qiagen) according to the manufacturer’s protocol. The RNA extraction from other sample materials, including the blood of animal C7 (direct from 300 μL blood frozen with an equal amount of RNAlater), followed the TRIzol reagent (Roche) protocol as described (10, 30). NGS library preparation from isolated RNAs, sequencing, and subsequent genome assembly were done as described (10, 30, 31).

### Phylogenetic analyses.

The amino acid sequences of previously identified hartmaniviruses were retrieved from GenBank. The MAFFT E-INS-i algorithm (41) was used for aligning the sequences with those of the viruses identified in this study. The Bayesian Monte Carlo Markov chain (MCMC) method implemented in MrBayes v3.2.6. (42) served for inferring the best-fit amino acid substitution models and constructing phylogenetic trees. In total, 500,000 generations with sampling at every 5,000 steps were run in MrBayes. The final standard deviations between two runs were <0.02 for all analyses.
